# Analysis of the Biological Properties of Blood Plasma Protein with GcMAF Functional Activity

**DOI:** 10.3390/ijms23158075

**Published:** 2022-07-22

**Authors:** Evgeniya V. Dolgova, Svetlana S. Kirikovich, Evgeniy V. Levites, Vera S. Ruzanova, Anastasia S. Proskurina, Genrikh S. Ritter, Oleg S. Taranov, Nikolay A. Varaksin, Tatiana G. Ryabicheva, Olga Yu. Leplina, Alexandr A. Ostanin, Elena R. Chernykh, Sergey S. Bogachev

**Affiliations:** 1 Institute of Cytology and Genetics of the Siberian Branch of the Russian Academy of Sciences, 630090 Novosibirsk, Russia; dolgova.ev@mail.ru (E.V.D.); svetak@bionet.nsc.ru (S.S.K.); levites@bionet.nsc.ru (E.V.L.); ruzanova@bionet.nsc.ru (V.S.R.); likhacheva@bionet.nsc.ru (A.S.P.); ritter@bionet.nsc.ru (G.S.R.); 2 Department of Natural Sciences, Novosibirsk National Research State University, 630090 Novosibirsk, Russia; 3 State Research Center of Virology and Biotechnology “Vector”, 630559 Koltsovo, Russia; taranov@vector.nsc.ru; 4 JSC “Vector-Best”, 630559 Koltsovo, Russia; varaksin@vector-best.ru (N.A.V.); tatria@mail.ru (T.G.R.); 5 Research Institute of Fundamental and Clinical Immunology, 630099 Novosibirsk, Russia; oleplina@mail.ru (O.Y.L.); ostanin62@mail.ru (A.A.O.); ct_lab@mail.ru (E.R.C.)

**Keywords:** vitamin D3 binding protein, group-specific component protein-derived macrophage-activating factor, M1/M2 macrophages, Lewis carcinoma

## Abstract

The main problem related to the studies focusing on group-specific component protein-derived macrophage-activating factor (GcMAF) is the lack of clarity about changes occurring in different types of macrophages and related changes in their properties under the effect of GcMAF in various clinical conditions. We analyzed the antitumor therapeutic properties of GcMAF in a Lewis carcinoma model in two clinical conditions: untreated tumor lesion and tumor resorption after exposure to Karanahan therapy. GcMAF is formed during site-specific deglycosylation of vitamin D3 binding protein (DBP). DBP was obtained from the blood of healthy donors using affinity chromatography on a column with covalently bound actin. GcMAF-related factor (GcMAF-RF) was converted in a mixture with induced lymphocytes through the cellular enzymatic pathway. The obtained GcMAF-RF activates murine peritoneal macrophages (*p* < 0.05), induces functional properties of dendritic cells (*p* < 0.05) and promotes in vitro polarization of human M0 macrophages to M1 macrophages (*p* < 0.01). Treatment of whole blood cells with GcMAF-RF results in active production of both pro- and anti-inflammatory cytokines. It is shown that macrophage activation by GcMAF-RF is inhibited by tumor-secreted factors. In order to identify the specific antitumor effect of GcMAF-RF-activated macrophages, an approach to primary reduction of humoral suppressor activity of the tumor using the Karanahan therapy followed by macrophage activation in the tumor-associated stroma (TAS) was proposed. A prominent additive effect of GcMAF-RF, which enhances the primary immune response activation by the Karanahan therapy, was shown in the model of murine Lewis carcinoma. Inhibition of the suppressive effect of TAS is the main condition required for the manifestation of the antitumor effect of GcMAF-RF. When properly applied in combination with any chemotherapy, significantly reducing the humoral immune response at the advanced tumor site, GcMAF-RF is a promising antitumor therapeutic agent that additively destroys the pro-tumor properties of macrophages of the tumor stroma.

## 1. Introduction

Group-specific component protein-derived macrophage activating factor (GcMAF), an important component of the macrophage activation system, is produced as a result of site-specific deglycosylation of vitamin D_3_ binding protein (DBP). DBP is converted to GcMAF through cleavage of galactose and sialic acid from N-Acetylgalactosamine (GalNAc) (Thr420) by *β*-galactosidase and sialidase residing on the cell membranes of activated B and T lymphocytes, respectively [1,2,3,4,5,6,7].

The interest in GcMAF is due to its ability to participate in the body’s defense mechanisms through macrophage activation: defense against pathogens, malignant, and damaged cells; as well as healing processes.

There are two subtypes of macrophages that are most clearly distinguished. They are designated as M1 and M2 cells exhibiting pro- and anti-inflammatory activity, respectively [8,9]. The functional status of macrophages was found to be largely determined by conditions of their activation and inactivation [7,10,11].

M1 macrophages play an important role in tumor cell elimination. They can exhibit cytotoxic, microbicidal, and antiproliferative activities mediated by the production of reactive oxygen species (e.g., H_2_O_2_), nitrogen monoxide (NO) metabolites, and pro-inflammatory cytokines.

M2 macrophages, on the contrary, exhibit anti-inflammatory activity and thus limit the inflammatory/immune response. Increased activity of M2 macrophages is associated with immunosuppression resulting in tumor growth [12,13,14].

Numerous reports for experimental animals and data obtained in preclinical studies indicate that it is promising to use GcMAF in clinical practice. Specific properties of the macrophage activator and the positive previous experience of its clinical use have underlain the clinical trials of the preparation against COVID-19 [15,16,17].

One of the most clinically relevant properties of GcMAF-activated macrophages is their antitumor activity [1,2,7,18,19,20,21,22,23,24,25,26].

Meanwhile, other results that do not confirm the effectiveness of GcMAF in clinical practice have led to strong skepticism about this factor in the scientific community [15,27,28,29]. 

An original approach to producing DBP and its conversion to GcMAF-related factor (GcMAF-RF) via a cytoenzymatic pathway was invented in the Laboratory of Induced Cellular Processes of the Institute of Cytology and Genetics of the Siberian Branch of the Russian Academy of Sciences in association with limited liability company (LLC) “ACTIVATOR MAF”.

We suggested that the main problem in GcMAF studies is the lack of clarity about changes occurring in different types of macrophages and related changes in their properties under the effect of GcMAF in various clinical conditions. In the current study, we analyzed the antitumor therapeutic properties of GcMAF in two clinical conditions: in untreated advanced tumors and during tumor resorption after exposure to the Karanahan approach. Incurable fast-growing Lewis carcinoma was selected as a model in the study.

The study consists of two integrated parts. It is aimed mainly at characterizing the antitumor effect of GcMAF-RF. In the first part, the ability of GcMAF-RF to activate various cell populations was characterized. We found that the preparation could induce M1 polarization of M0 phagocytes under ex vivo conditions. We also showed that GcMAF-RF-activated blood cells produce both pro- and anti-inflammatory cytokines. These two findings have led to the following conclusions: (1) the macrophage phenotype can be altered by exposure to GcMAF-RF under certain conditions, and (2) the activator will enhance the properties of the type of macrophages that is supported by the existing humoral background (M1/M2). This is due to the simultaneous secretion of both pro- and anti-inflammatory cytokines by activated GcMAF-RF cells, which will affect the existing phagocyte phenotype.

The second part includes a comprehensive analysis of antitumor responses mediated by the action of GcMAF-RF. In this part of the study, evidence was found for the hypothesis that GcMAF-RF activates properties of the existing macrophage phenotype and, in particular, tumor-associated M2 phagocytes. In addition, it turned out that the humoral background of tumor-associated stroma can lead to complete anergy of GcMAF-RF-activated macrophages towards the M1 phenotype. This observation was confirmed by numerous unsuccessful attempts to detect the antitumor effect of GcMAF-RF upon injection to the tumor lesion when using monotherapy. We suggested that, once the dominant effect of the cytokine background produced by the tumor lesion is eliminated, the treatment of macrophages of the tumor-associated stroma would change the pro-tumor M2 phenotype to neutral M0 or antitumor M1 phenotypes, which would result in pronounced tumor regression.

A novel approach for treating oncological diseases known as “Karanahan” (derived from the Sanskrit “killing the source”) has been under development in the Laboratory of Induced Cell Processes of the Institute of Cytology and Genetics of the Siberian Branch of the Russian Academy of Science (Novosibirsk, Russia). The therapy is a universal approach to treating cancer, based on eradicating cancer stem cells (CSCs). The technology is based on three discoveries made in the laboratory:

1. Discovery of a feature that is universal for poorly differentiated tumorigenic cancer cells (CSCs), namely the ability to internalize fragments of extracellular double-stranded DNA (dsDNA) through the natural mechanism, and the discovery of the TAMRA-labeled DNA probe that is used as a specific molecular marker of CSCs [30,31].
2. Discovery of the ability of these DNA fragments to interfere with interstrand crosslink repair when delivered to intracellular compartments, leading to either CSC death or loss of their tumorigenic properties. Once having lost its basis for infinite existence, the tumor is eliminated by the defense systems [30,32].
3. Discovery of the mechanism of CSC synchronization in a specific tumor at the treatment-sensitive phase of the cell cycle and application of identified time profile to eradicate the tumorigenic origin, namely, CSCs [32].

The approach consists of the following procedures:

1. Detection of poorly differentiated CSCs in a tumor based on their capability to internalize the TAMRA-labeled dsDNA probe.
2. Identification of the time profile of interstrand crosslink repair cycle induced by a crosslinking cytostatic agent.
3. Triple cell cycle arrest of committed and cancer stem cells in the G2/M phase based on identified parameters of the time profile of the repair cycle followed by determination of the day of synchronous exit of TAMRA+ CSCs and their accumulation in the therapy-sensitive G1 phase.
4. Injection of a composite dsDNA preparation at the point of demarcation between the phases of nucleotide excision repair (NER) and homologous recombination (HR) in such a way that each component of a therapeutic agent interferes with either NER or HR. This completely deprives a tumor-initiating stem cell of its ability to survive the therapeutic “assault” leading to either cell death or loss of its tumorigenic potential. In addition, the simultaneous administration of the composite dsDNA preparation induces massive apoptosis of committed cancer cells.
5. A composite dsDNA preparation developed in the laboratory is used as a therapeutic agent. The development of the approach is described in detail in [33].

A pronounced antitumor effect of the therapy was demonstrated in several experimental models of incurable fast-growing cancers [32,33,34,35,36].

We hypothesized that, during the response to Karanahan therapy in tumor-bearing mice, the tumor lesion under treatment would stop secreting pro-tumor factors (cytokines) and form a pro-tumor humoral background at a certain point of regression. It is the time point when tumor-associated M2 macrophages lose their cytokine support of the pro-tumor phenotype and become an accessible target for GcMAF-RF. This is the approach that was used in the study for determining the conditions under which GcMAF-RF-activated macrophages would unambiguously exhibit the antitumor effect.

## 2. Results

### 2.1. GcMAF-RF Preparation

As stated above, DBP contains three functional sites: the vitamin D3 binding domain, the actin-binding segment in the polypeptide chain, and the glycosylation site. Hence, there are two obvious ways of affinity isolation of the protein: via either the vitamin D3 binding domain or the actin-binding domain. Other authors almost always present the same method of obtaining the macrophage activator GcMAF [37,38]. Vitamin D_3_ is enzymatically converted into a derivative molecule containing a hydroxyl group at position 25. After the modified vitamin is bound to cyanogen bromide-activated Sepharose, affinity chromatography is performed.

As mentioned, DBP contains one trisaccharide at position 420 of a threonine residue. The trisaccharide consists of GalNAc with two branched sugar residues: galactose and sialic acid. Elimination of these two sugars is the necessary stage of DBP conversion to GcMAF. DBP is converted to GcMAF with a series of modifications. The oligosaccharide is hydrolyzed by membrane-associated enzymes β-galactosidase and sialidase, which are activated by the inflammatory process triggered by B and T cells. The β-galactosidase enzyme resides on the cytoplasmic membrane of B cells, and sialidase is found on the membrane of T cells, which bind to DBP through specific receptors. As a result of these modifications, an active protein (GcMAF) containing a GalNAc residue is formed. The presence of GalNAc is a necessary condition for the manifestation of the functional activity of GcMAF [1,25]. 

The functional activity of the resulting activated factor is tested based on its ability to activate peritoneal macrophages (PMs) to phagocytose various extracellular particles. Opsonized sheep erythrocytes are mainly used for this purpose [1,39].

In this study, we developed a protocol for obtaining a large amount of DBP and converting it to GcMAF-RF (see “GcMAF-RF preparation”). The method using metal beads was validated to assess the phagocytic activity of the resulting GcMAF-RF preparation [40]. The procedure is well standardized; it does not require multistage production of opsonized sheep erythrocytes and is high-end technology. To assess the specificity of isolated GcMAF-RF, an original method for obtaining lectin was developed. The method makes it possible to assess the efficiency of sugar tail cleavage and preserve GalNAc, which is the key molecule involved in macrophage activation, at the glycosylation site (industrial property of LLC “ACTIVATOR MAF”, Novosibirsk, Russia).

**Figure 1:**
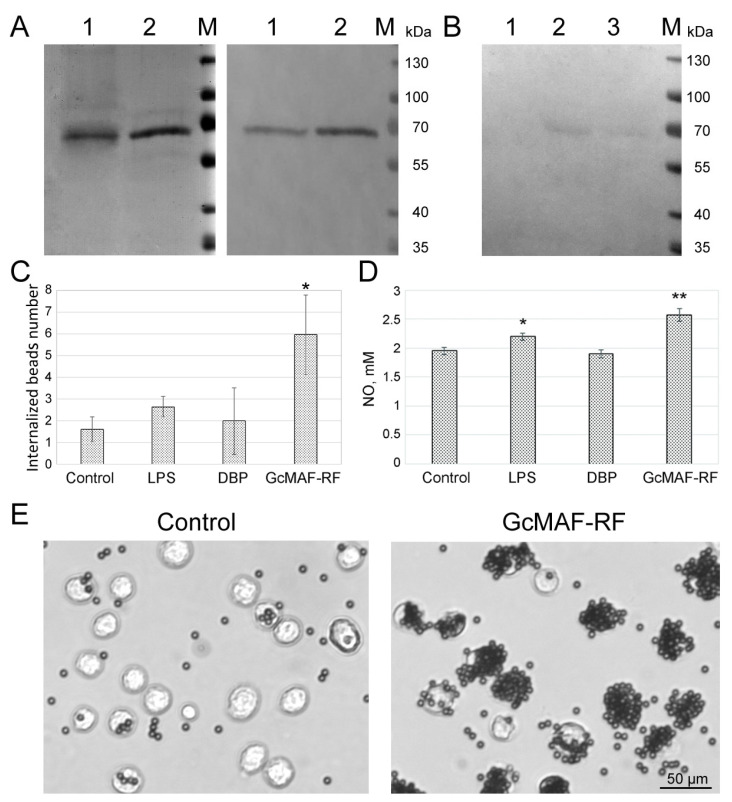
Characterization of DBP and DBP-derived GcMAF-RF. (**A**) Polyacrylamide gel of DBP samples obtained using actin-agarose (1), and Sepharose (with 25-hydroxyvitamin D_3_) (2) affinity chromatography (left) and Western blot of these samples with antibodies against Gc group proteins (right). (**B**) Western blot of a polyacrylamide gel for *Helix pomatia* lectin. 1—DBP, 2—GcMAF-RF, and 3—FBS. M—Thermo ScientificTM Page RulerTM Prestained protein Ladder molecular marker (Thermo Fisher Scientific Inc., Waltham, MA, USA). (**C**) Phagocytic activity of murine PMs treated with LPS, DBP, and GcMAF-RF compared to control untreated macrophages. Data are presented as Mean ± SD (*n* = 4), * differences compared to the control are significant, *p* < 0.05 (Mann–Whitney *U* test). (**D**) NO production by murine PMs treated with LPS, DBP, and GcMAF-RF compared to control untreated macrophages. Data are presented as Mean ± SD (*n* = 5), differences compared to the control are significant, * *p* < 0.05, ** *p* < 0.01 (Mann–Whitney *U* test). (**E**) Images of the beads phagocytized by naïve macrophages (control) and macrophages exposed to GcMAF-RF.

Comparative western blot analysis was performed to verify the correspondence of DBP obtained using actin as an affinity ligand to DBP isolated by an affinity for 25-hydroxyvitamin D_3_ Sepharose and determine whether the polypeptide belongs to the group of specific Gc proteins [3,4]. Analysis of the samples together with antibodies against the Gc group proteins indicates the identity of the GcMAF-RF proteins obtained by two different methods of affinity chromatography (Figure 1A,B). Additional affinity chromatography and macrophage activation were performed to confirm that it is the converted GcMAF-RF rather than lymphocyte metabolites that increase the phagocytic activity of the drug [41].

An analysis of the production of open-chain GalNAc, which is an indicator of deglycosylation, revealed that FBS used for culturing also contained a fraction reacting with the specific ligand *Helix pomatia* lectin after the conversion of DBP to GcMAF-RF. However, the intensity of interaction with *Helix pomatia* was twice as high in the GcMAF-RF sample than in the FBS sample (Figure 1B). This fact indicated that the GcMAF-RF sample contained GalNAc, which was opened as a result of treatments. Additional comparative experiments were conducted to test the involvement of the FBS protein carrying a free sugar residue in the activation of phagocytic activity of macrophages. FBS was shown to have no effect on the phagocytic activity of PMs. The obtained results also indicate that non-converted DBP does not interact with *Helix pomatia* (Figure 1B). The molecular characteristics of the specific macrophage activator GcMAF-RF correspond to GcMAF described in the literature.

After generation, conversion, and verification of the specific ability to activate the phagocytic activity of macrophages, the GcMAF-RF preparation was sterilized by filtration.

### 2.2. Assessment of Phagocytic Activity and Ability of PMs Treated with GcMAF-RF to Secrete Nitric Oxide

The phagocytic activity of macrophages was assessed by internalization of magnetic beads [40] and calculated using such parameters as the internalized beads number (IBN), which indicates the ratio between the number of internalized beads and the total number of macrophages 3 h after the exposure to activators (see further details in “Materials and Methods” section). Naïve intact macrophages were used as a negative control; macrophages exposed to 10 μg/mL LPS served as a positive control. Figure 1C shows a histogram indicating the specific activity of the compound. Each batch of GcMAF-RF preparation was tested for its ability to induce the phagocytic activity of PMs. A 3–7-fold increase in phagocytic activity was observed depending on the macrophage donor animal.

GcMAF enhanced not only the phagocytic activity of PMs but also the production of nitric oxide (NO) by them. Figure 1D illustrates a statistically significant (*p* < 0.01) increase in NO production by macrophages exposed to GcMAF (but not to its precursor DBP). Moreover, GcMAF-induced NO production was even higher than that induced by the standard macrophage activator LPS. Figure 1E illustrates the ability of GcMAF-RF-treated murine PMs to phagocytize magnetic beads.

### 2.3. Ex Vivo Effects on Human Immunocompetent Cells

#### 2.3.1. Activation of Functional Properties of Dendritic Cells by GcMAF-RF

**Figure 2:**
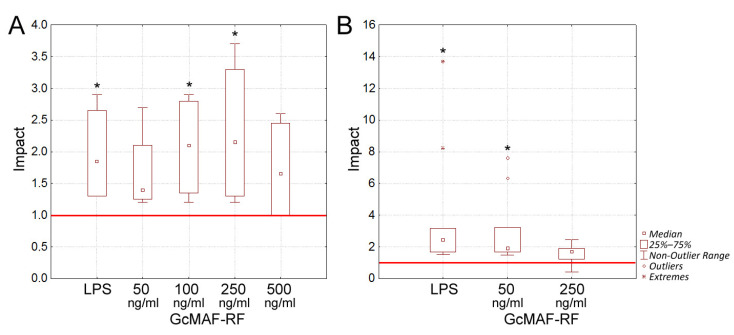
Ex vivo analysis of the effect of GcMAF-RF on immunocompetent cells. (**A**) Allostimulatory activity of human DCs activated by either the standard activator LPS or various concentrations of GcMAF-RF. The allostimulatory activity of DCs in the absence of activators was taken to be unity (red line). * significant differences from DCs without activators, *p* < 0.05 (Mann–Whitney *U* test). (**B**) The effect of GcMAF-RF on M1 polarization of human macrophages. Polarization was assessed based on allostimulatory activity of M1 polarized macrophages. LPS and GcMAF (50 and 250 ng/mL) were used as maturation inducers. Allostimulatory activity of intact macrophages was taken to be unity (red line). * significant differences from unstimulated macrophages, *p* < 0.01 (Mann–Whitney *U* test). Impact—the ratio between stimulated DCs or macrophages and intact ones.

The stimulatory effect of GcMAF-RF on in vitro maturation and function of human dendritic cells (DCs) was evaluated. DCs were obtained from peripheral monocytes and cultured in the presence of either LPS (positive control) or various concentrations of GcMAF-RF (50, 100, 250, and 500 ng/mL of the target protein) for 24 h. Intact DCs incubated without any inducer were used as a negative control. The allostimulatory activity of DCs (i.e., their ability to stimulate proliferation of allogeneic T lymphocytes) treated with LPS was compared to that of DCs treated with GcMAF-RF. The proliferative response of lymphocytes was estimated by measuring the incorporation of ^3^H-thymidine (1 μCi/well). The experiment was conducted in DCs obtained from four donors. The allostimulatory activity is shown as the impact index (II), which is the ratio between stimulated and intact DCs (Figure 2A). II exceeding 1 indicates an increase in allostimulatory activity compared to the control (intact DCs).

The results indicate that the allostimulatory activity of DCs increased in response to GcMAF-RF exposure during maturation, and this increase was comparable to that observed after exposure to LPS (*p* < 0.05). The most prominent effect of GcMAF-RF was detected at the dose of 250 ng/mL (II ranged from 1.2 to 2.9, *p* < 0.05). The stimulatory effect of GcMAF-RF similar to that of LPS was achieved at a concentration of 100 ng/mL.

Thus, GcMAF-RF at concentrations of 100 and 250 ng/mL was shown to activate the maturation of DCs from four independent donors in four independent experiments.

#### 2.3.2. M1 Polarization of M0 Macrophages by GcMAF-RF

We assessed the stimulatory effect of GcMAF-RF on M1 polarization of GM–CSF-treated macrophages in vitro.

LPS is known to stimulate the polarization of macrophages to the M1 phenotype, which plays an important role in anti-tumor defense [42]. One of the functional hallmarks of M1 cells is their high allostimulatory activity. In this study, we performed a comparative assessment of the effects of GcMAF-RF and LPS on the allostimulatory activity of GM–CSF-differentiated (unpolarized) macrophages.

Peripheral blood monocytes were grown in the presence of GM–CSF and further exposed to either LPS or GcMAF-RF (50 and 250 ng/mL) for 48 h. Intact GM–CSF-maturated mononuclear cells (MNCs) were used as a negative control. Macrophages were cultured with allogeneic MNCs for evaluating their allostimulatory activity. The proliferative response was estimated on day 5 by measuring the incorporation of ^3^H-thymidine (1 μCi/well). GM–CSF-maturated macrophages from three different donors and MNCs from three other different donors were used in the experiments. As a result, nine independent combinations were obtained. The allostimulatory activity is shown as the II, which is the ratio between the number of stimulated macrophages and that of non-stimulated ones (Figure 2B).

The obtained data indicate that the allostimulatory activity of unpolarized macrophages is elevated after their exposure to M1-inducing LPS (positive control). GcMAF-RF had a similar effect.

Thus, similarly to LPS, GcMAF-RF elevates the allostimulatory activity of macrophages, which indicates its ability to induce M1 polarization of naïve macrophages.

#### 2.3.3. Cytokine Production by Whole Blood Cells of Healthy Donors in Response to GcMAF-RF Stimulation

To assess the effect of GcMAF-RF on cytokine production by human whole blood cells, blood freshly isolated from healthy donors was treated with different concentrations of a macrophage activator (three donors, two separate isolates of GcMAF-RF, a total of six variants). We analyzed the cytokines belonging to pro-inflammatory and anti-inflammatory groups, growth factors, and chemokines. Constitutive cytokine production was assessed in the absence of activators and after activation by various concentrations of GcMAF-RF.

**Figure 3:**
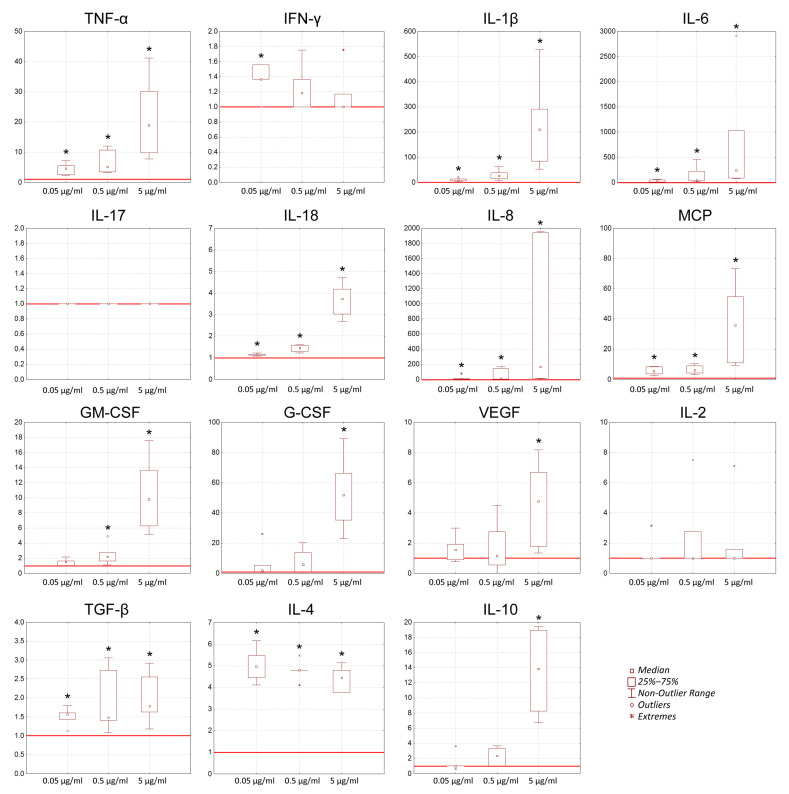
Induction of cytokine production by GcMAF-RF-activated whole blood cells of healthy donors. The Y axis represents the impact index: the ratio between cytokine production after exposure to GcMAF-RF at a certain concentration and constitutive cytokine production taken as unity (red line). * significant differences in cytokine production after exposure to GcMAF-RF relative to constitutive cytokine production (*n* = 6), *p* < 0.05 (Mann–Whitney *U* test).

The addition of GcMAF-RF to the whole blood stimulated the production (pg/mL) of early-phase inflammatory cytokines and other pro-inflammatory cytokines (TNFα, IFN-γ, IL-1β, IL-6, IL-17, and IL-18) by the cells (Figure 3). The increase in the level of these cytokines, except for IL-17 and IFN-γ, exceeded the constitutive cell activity 2–3000 times. The chemokines IL-8 and MCP also demonstrated high levels, exceeding those of the constitutive activity by 10–2000 times. The levels of growth factors GM-CSF, G-CSF, and VEGF were increased 2–90-fold compared to constitutive production. A 2–20-fold increase is shown in the group of anti-inflammatory cytokines (TGF-β, IL-4, IL-10) compared to the group with constitutive production. A pronounced dose-dependent effect was observed for most cytokines. This means that injections of GcMAF-RF stimulated simultaneous secretion of functionally opposite cytokines, thus causing both the inflammatory and anti-inflammatory responses (or pro-tumor and antitumor responses in the case of tumor) (Figure 3).

### 2.4. Evaluation of the Antitumor Effect of GcMAF-RF

#### Assessment of the Direct and Macrophage-Mediated Cytotoxic Effects of GcMAF-RF on U87 and MCF-7 Tumor Cells Ex Vivo

Two tumor cell lines were used to determine the cytotoxic effect of GcMAF-RF: human breast adenocarcinoma MCF-7 and human glioblastoma U87 cells.

**Figure 4:**
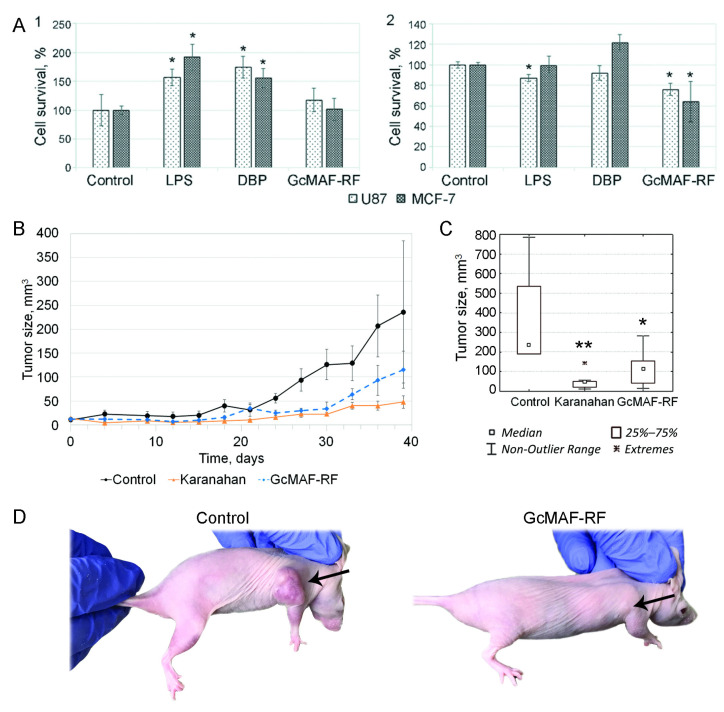
The antitumor effect of GcMAF-RF. (**A**) The cytotoxic effect of GcMAF-RF on U87 and MCF-7 cancer cells. **1**—direct cytotoxicity; **2**—PM-mediated cytotoxicity. Mean ± SD values are shown (*n* = 3); * *p* < 0.05—significance of differences compared to the control (Mann–Whitney *U* test). (**B**–**D**) The effects of Karanahan therapy and GcMAF-RF administration alone on human U87 glioblastoma inoculated subcutaneously in immunodeficient mice. (**B**) Graph demonstrating growth of a human U87 glioblastoma graft. Mean ± SEM values are shown. (**C**) Graph showing graft volumes at the terminal stage (39 days after therapy initiation). Significance of differences (* *p* < 0.05; ** *p* < 0.01, Mann–Whitney *U* test). (**D**) Photographs of control group mice and animals receiving GcMAF-RF, 39 days after therapy initiation. Arrows indicate tumors.

GcMAF-RF had no direct cytotoxic effect on tumor cells. The exposure of these cells to LPS and DBP increased their survival (*p* < 0.05) (Figure 4(A1)). Co-culturing of MCF-7 and U87 cells with PMs pretreated with GcMAF-RF reduced the viability of both cell lines by 25–35% (*p* < 0.05) (Figure 4(A2)).

### 2.5. Evaluation of the Antitumor Effect of GcMAF-RF In Vivo

Because of the observed property of GcMAF-RF, i.e., the ability to polarize M0 macrophages to the M1 phenotype, we hypothesized that intratumoral injection of GcMAF-RF would reactivate M2 macrophages of the tumor-associated stroma (TAS) to the tumor-reactive M1 phenotype and facilitate the lysis of the tumor graft. Several series of experiments were performed on various cancers based on this assumption [43].

GcMAF-RF was shown to exhibit functionally opposite effects on tumor graft progression when used alone. Repeated attempts to detect the antitumor effect of GcMAF-RF on Krebs-2 (ascites, solid tumor, initial graft size of 0.2 cm^3^) and Lewis (solid intramuscular graft, initial graft size of 0.2 cm^3^) carcinoma grafts during intratumoral injections of GcMAF-RF and on U87 (solid subcutaneous graft, initial graft size of 0.2 cm^3^) cells upon intraperitoneal/intratumoral injections of GcMAF-RF were not successful. In all the described cases, changes in tumor growth in the group receiving GcMAF-RF only were identical to those in the control group receiving saline injections and even stimulated graft growth in some cases. In other words, GcMAF-RF injections had no suppressive effect on tumor growth (data not shown). The antitumor effect of GcMAF-RF administered alone was observed following alternating intraperitoneal and intratumoral injections in the case of subcutaneous U87 graft with an initial tumor graft volume of 0.02 cm^3^ (Figure 4B–D).

An analysis of the entire dataset obtained might have indicated that the drug used alone cannot induce repolarization of M2 tumor-associated macrophages to the M1 tumor-reactive state in developed tumors. This process is assumed to be inhibited by the humoral action of the tumor. 

### 2.6. Combining Two Approaches, In Situ Vaccination with GcMAF-RF and Karanahan Therapy, in One Therapeutic Procedure

**Figure 5:**
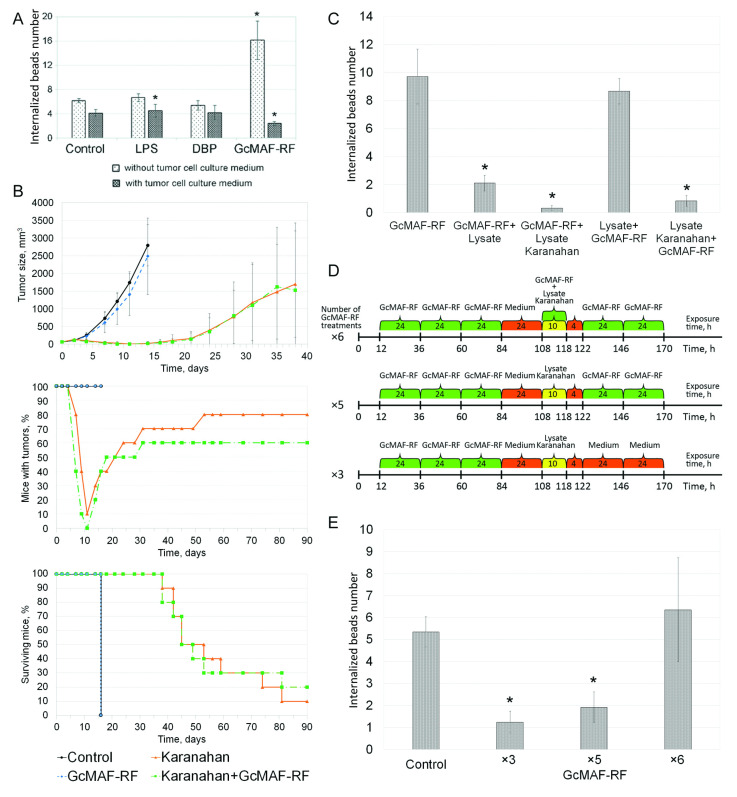
Analysis of antitumor properties of GcMAF-RF. (**A**) Effect of tumor cell culture medium on the ability of GcMAF-RF to activate murine PMs. Mean ± SD values are shown (*n* = 4). * significant differences from the corresponding control, *p* < 0.05 (Mann–Whitney *U* test). (**B**) Effects of the Karanahan therapy alone and Karanahan therapy in combination with GcMAF-RF in mice with Lewis carcinoma. Mean ± SD values are presented. (**C**) Loss of specific phagocytic activity by murine PMs due to treatment with tumor lysate. Macrophages were either treated with GcMAF-RF alone, GcMAF-RF and Lewis carcinoma tumor lysate, GcMAF-RF and Lewis carcinoma tumor lysate obtained from mice on day 7 after Karanahan therapy, or first exposed to corresponding tumor lysates and then activated by GcMAF-RF. PM pretreatment scheme is given in Supplementary Data S2, Figure S3. The values of PM activation indices (Mean ± SD) are presented. * significant differences from GcMAF-RF, *p* < 0.05 (Mann–Whitney *U* test). (**D**) Schematic representation of the search for regimens of PM treatment. (**E**) Phagocytic activity of PMs in the absence of additional treatments (control), treated with GcMAF-RF three, five, or six times with a 24-h interval, and treated with a Lewis carcinoma lysate obtained from mice on day 7 after the Karanahan therapy. The values of PM activation indices (Mean ± SD) are shown. * significant differences from the control, *p* < 0.05 (Mann–Whitney *U* test).

Experiments on treating GcMAF-RF-activated murine PMs with a conditioned medium from a culture of human B lymphoma cells indicate the possibility of inhibiting macrophage activity by factors secreted by tumor cells (Table 1; Figure 5A).

It turned out that activated GcMAF-RF macrophages lose their phagocytic activity, which is one of the main indicators of the M1 phenotype of phagocytes, as a result of the action of conditioned medium factors [44]. We can assume that IL-8, MCP, or VEGF of the conditioned medium induced macrophage anergy.

The obtained ambiguous results on the antitumor activity of GcMAF-RF and direct confirmation of inhibition of phagocytic activity by GcMAF-RF-activated conditioned media from a tumor cell culture indicated that TAS actively counteracts the interaction between GcMAF-RF and tumor-associated macrophages.

We have put forward a hypothesis that the antitumor effect of GcMAF-RF can be induced only in the case of a significant reduction in the humoral activity of the tumor. Based on the data obtained, we decided to combine two approaches: in situ vaccination with GcMAF-RF and Karanahan therapy [35,45]. We hypothesized that the cytoreductive effect of the Karanahan approach would enable the antitumor properties of GcMAF-RF used in the in situ vaccination regimen. The Karanahan approach would induce massive apoptosis of committed tumor cells and eradication of tumor stem cells. In situ vaccination with GcMAF-RF would activate macrophages free of humoral tumor burden, which would activate an antitumor immune response associated with either the activity of macrophages repolarized to the M1 phenotype or inhibition of the activity of tumor-associated M2 cells.

#### Searching for the Conditions Enabling GcMAF-RF Antitumor Activity and Their Characterization

The initial experiments to test the new hypothesis of activating the antitumor properties of GcMAF-RF in combination with the Karanahan therapy showed that the latter enables the antitumor properties of GcMAF-RF in the Lewis carcinoma model. In this model, no antitumor effect was previously obtained under any circumstances. Multiple intratumoral injections of GcMAF-RF showed positive effects on solid tumors of weakly immunogenic Lewis carcinoma (Figure 5B).

We searched for the time period during Karanahan therapy when pronounced antitumor properties of GcMAF-RF could be manifested. We isolated and analyzed Lewis carcinoma lysates following Karanahan therapy. We selected lysates obtained on therapy day nine, when the tumor was reduced significantly, for analysis. It turned out that treatment with tumor lysate isolated after the Karanahan therapy triggers irreversible pronounced anergy of murine PMs. A single treatment with GcMAF-RF either before or after incubation with tumor lysates did not restore macrophage activity (Figure 5C).

**Figure 6:**
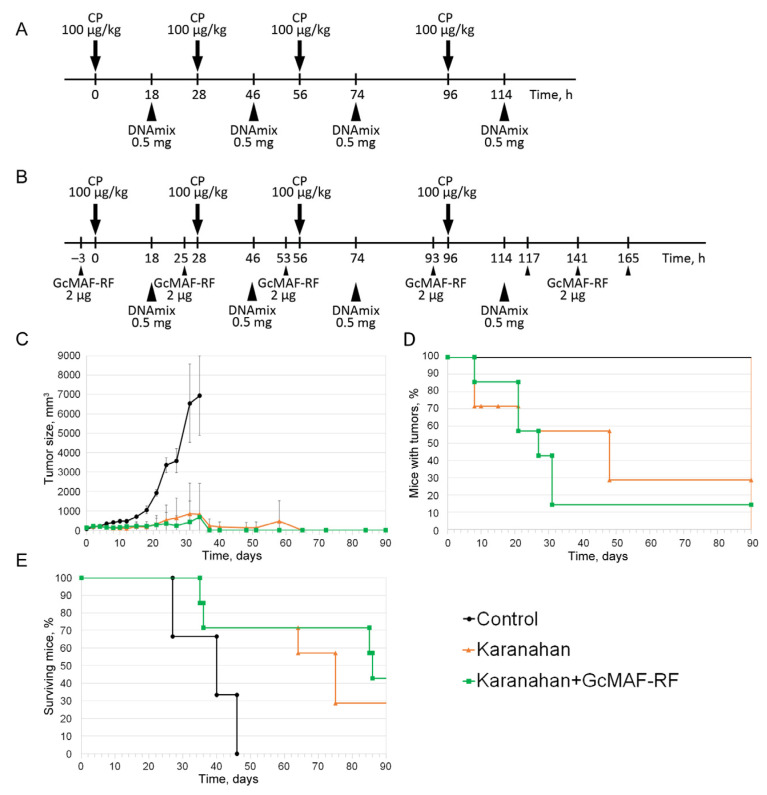
Comparison of the antitumor efficacy of the Karanahan approach alone and in combination with intratumoral injections of GcMAF-RF in the model of weakly immunogenic Lewis carcinoma. (**A**) Therapeutic regimen for treating Lewis carcinoma using the Karanahan approach. (**B**) Therapeutic regimen for treating Lewis carcinoma using the Karanahan approach in combination with intratumoral injections of GcMAF-RF. Arrows indicate intraperitoneal injections, arrowheads—intratumoral. (**C**) Changes in tumor growth in the study and control groups. Mean ± SD values are shown. (**D**) Changes in the number (%) of mice with tumors. (**E**) Animal survival graph.

Since initial in vivo experiments showed that GcMAF-RF can restore the functional activity of macrophages when used in combination with the Karanahan therapy, macrophages were massively treated with GcMAF-RF at various time points together with the use of Karanahan therapy. A regimen that made it possible to apply the antitumor properties of the Karanahan approach (lysates of resorbed tumor, seven days of therapy) while maintaining the viability and inducing the phagocytic (antitumor) activity of PMs was found (Figure 5D,E). This treatment regimen showed that multiple treatments with GcMAF-RF are required during the course of the Karanahan therapy. This approach was applied in in vivo experiments (Figure 6).

Abundant purulent discharge from the treated paw (presumably neutrophilic inflammation caused by neutrophil attraction to the tumor growth site) was noted on day 15 to 27 in some mice. Tumors disappeared completely after the formation of purulent discharge in the Karanahan + GcMAF-RF Group, while in the Karanahan Group, the tumor progressed after the occurrence of purulent discharge, which led to animal death (Figure 6E).

### 2.7. Evaluation of the Immune Response Induced by Karanahan Monotherapy and Karanahan Therapy in Combination with In Situ Vaccination with GcMAF-RF in Mice with Lewis Carcinoma

A pilot study (primary immunogram) was conducted in the first stage. The study was aimed at assessing the enhancement of the immune responses by Karanahan therapy [46], which is assumed to mediate a significant positive effect of GcMAF on the survival of tumor-bearing mice, by including GcMAF-RF in the treatment regimen (Figure S1, Supplementary Data S1).

The main goal of the approach was to determine the time of onset of the immune response and its direction. In addition, this analysis revealed that residual tumor does not affect changes in the analyzed populations. The main factor responsible for these changes is time after therapy (Figure S1, Supplementary Data S1).

**Figure 7:**
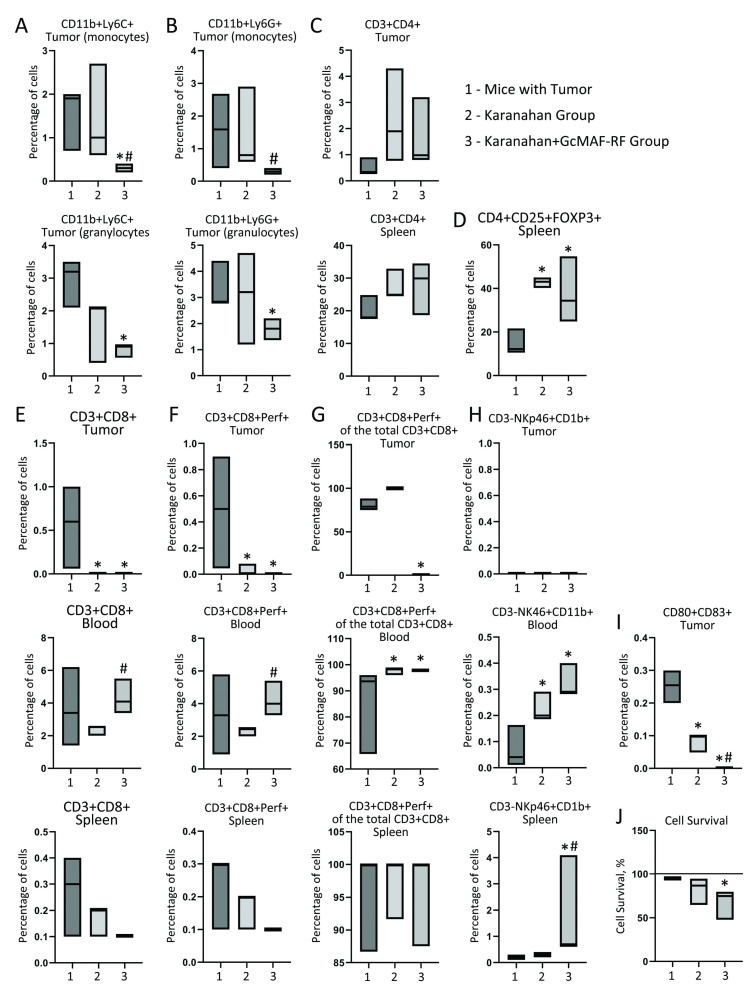
The additive effect of GcMAF on primary induction of the immune response by Karanahan therapy in mice with Lewis carcinoma on day 16. (**A**) CD11b + LyC + cells. (**B**) CD11b + LyG+ cells. (**C**) CD3 + CD4 + cells. (**D**) CD4 + CD25 + FOXP3+ cells. (**E**) CD3 + CD8 + cells. (**F**) CD3 + CD8 + Perf + killer T cells. (**G**) CD3 + CD8 + Perf + cells among CD3 + CD8 + cells. (**H**) CD3-NKp46 + CD11b + cells. (**I**) CD80 + CD83 + cells. The black asterisk shows a significant difference between the control and experimental groups. The hash stands for a significant difference between the experimental groups. The confidence level is *p* < 0.05 (Mann–Whitney *U* test, *n* = 3). (**J**) Cytolytic activity of PMs against Lewis carcinoma cells. Median (line) with interquartile range (box) are shown.

A similar study aimed to confirm the results obtained in the pilot study (primary immunogram), and assess the statistical significance of detected changes in the analyzed cell populations at the second stage. According to the primary immunogram, the main changes in the analyzed cell populations were observed on days 15–17 (Figure S1). Because of that, we chose day 16 from the experiment initiation as a time point for analysis. This was the time point (day 15–17) when the first sites of necrotic tumor lysis with purulent discharge were observed, indicating an active catabolic process in the tumor. Tissues and organs from the control, Karanahan therapy, and Karanahan approach plus GcMAF-RF Groups were isolated on a selected day, and cell populations were analyzed as described in the pilot study. The results are presented in Figure 7. The description of cell populations shows the data characterizing changes in Karanahan + GcMAF-RF mice in comparison with Karanahan Group animals.

- MDSCs. There were significant differences in the primary immunograms. For both analyzed populations (monocyte and granulocyte fractions), a statistically significant reduction in the population of CD11b + LycC + and CD11b + LycG + cells, characteristic of MDSCs, was detected in the Karanahan + GcMAF Group (Figure 7A,B).
- T helper cells. The pattern of cell distribution did not differ from that in the primary immunogram recorded on day 17 of the experiment (Figure 7C).
- Regulatory T lymphocytes. The data obtained in the repeated experiment coincided with the results of the primary immunogram (Figure 7D).
- CD8 + Perf + cytotoxic T lymphocytes. The results of the repeated experiment for MNC samples were consistent with the results of the primary immunogram, which showed that GcMAF stimulates an increase in the number of cytotoxic lymphocytes in peripheral blood (Figure 7E,F). In addition, almost all CD3 + CD8 + cells were cytotoxic lymphocytes in the mononuclear fraction of treated groups (Figure 7G).
- Natural killer cells. An increase in the number of NK cells in the mononuclear fraction compared to the control was observed in the repeated experiment (Figure 7H). A significant rise in the number of NK cells was also noted in the spleen. The primary analysis showed a similar increase in the number of NK cells on day 15 of the experiment.
- DCs. The DC population was also assessed (Figure 7I). The number of functional antigen-presenting cells decreased in both experimental groups.

Thus, one can see that all the major patterns identified in the pilot study were observed repeatedly in most of the analyzed cell populations.

#### Evaluation of PM Cytolytic Activity

The cytolytic activity of PMs isolated from mice subjected to the Karanahan and Karanahan + GcMAF therapies and then co-cultured with Lewis carcinoma cells was evaluated using the MTT assay. Initial experiments showed that the number of living cells in a co-culture of tumor cells and PMs isolated from intact and tumor-bearing mice corresponded to the total number of living cells in individual samples of PMs and tumor cells. A similar experiment involving PMs from the Karanahan + GcMAF Group revealed that the percentage of viable cells were significantly decreased. PMs are terminally differentiated cells, and their cell number cannot be increased by division. The number of tumor cells can be increased in the sample due to their proliferative activity. These results suggest that PMs activated by the Karanahan + GcMAF therapy lyse Lewis carcinoma cells (Figure 7J). This conclusion was also confirmed by cytological analysis, which showed a decrease in the number of floating tumor cells.

## 3. Discussion

Numerous experimental studies conducted using various purified GcMAF preparations and activated plasma (the so-called second-generation GcMAFs) have indicated the diversity of biological effects of this factor [7,47,48]. The majority of these investigations were aimed at studying the anti-tumor activity of GcMAF. This activity was repeatedly shown to be mainly mediated by macrophage activation [5,25,49].

In the current study, a GcMAF-RF preparation purified from human serum using a new protocol developed by LLC “ACTIVATOR MAF” was used as a macrophage activating factor. The methods of drug purification utilize ligand affinity to specific functional domains of vitamin D_3_ binding protein [4,50,51,52]. The preparation activates the phagocytic and nitric oxide-producing activities of murine PMs, which are crucial in validating the active form of GcMAF.

The GcMAF-RF preparation has also been shown to activate the functional properties of another type of antigen-presenting cells, namely DCs. The preparation was shown to induce DC maturation and enhance their allostimulatory activity, and this effect was comparable to that of LPS, the standard inducer of DC maturation. The peak effect was observed at a dose of 250 ng/mL (*p* < 0.05). A decline in activating ability of GcMAF-RF with increasing preparation concentration is characteristic of substrate inhibition in an enzymatic assay. Apparently, at high concentrations, GcMAF can bind to a second site on macrophages (weak binding), where it inhibits the activating effect of GcMAF bound to the primary site (strong binding) [53].

We have also discovered a previously unknown property of the macrophage activating preparation: exposure of human M0 macrophages to 50 ng/mL GcMAF-RF induces their polarization towards the M1 phenotype.

An analysis of whole blood cell stimulation with GcMAF-RF yielded a surprising result. It induced the secretion of both pro-inflammatory cytokines, mainly TNFα and IL-1, which trigger inflammation, and a major anti-inflammatory cytokine IL-10.

The main goal of the study was to determine the conditions under which GcMAF-RF would have an unconditional anti-cancer effect. A direct ex vivo cytotoxicity assay showed that the preparation had no significant effect on tumorigenic MCF-7 and U87 cells in vitro. However, murine PMs activated by GcMAF exposure exhibited high cytotoxicity against both cell lines (MCF-7 and U87) and induced the death of up to 35% of tumor cells. In addition to the data on the polarization of M0 macrophages to the tumor-reactive M1 state, the data on murine macrophage activation by GcMAF-RF suggested the possibility of inducing in tumor repolarization of macrophages to the M1 phenotype by injecting the macrophage activator in the tumor, which should be accompanied by tumor destruction. However, contradictory results were obtained, which were explained by the following fact: activation actually occurs, but only for the state in which macrophages exist at the instant of activation. 

The results of our studies (unpublished data) indicate that GcMAF-RF selectively activates certain properties of macrophages, with the diversity of activities increasing due to the effect on different macrophages and other immune cells. We found that growth in the phagocytic activity might be accompanied by increased TGF-β and arginase production. This means that a decrease in pro-inflammatory properties and, as a consequence, the absence of T helper cell activation takes place when the phagocytic activity of GcMAF-RF-treated macrophages increases. One of the explanations for this effect is macrophage plasticity or the existence of several types of phagocytes (or other cells) with different properties that manifest themselves upon exposure to GcMAF-RF in the TAS. This hypothesis can be confirmed by points presented in recent studies [53,54].

In the case of isolated cell lines, GcMAF-RF activation stimulates the cellular response that is typical of their biological function under conditions of artificial activation and not quenched by the activity of other cell populations.

In the case of whole blood, various cell populations activated by GcMAF-RF secrete cytokines typical of their biological function, which induce diametrically opposed cellular responses as has been shown experimentally.

The tumor is known to be infiltrated by various groups of immune cells. All of them are activated according to their inherent characteristics upon GcMAF-RF injection into the tumor. The cells secrete functionally opposite cytokines. The antitumor response can be detected only in the case when cytokines inducing the antitumor cellular responses prevail. This is possible in the case of inhibition of the suppressor activity of TAS [55].

A reliable anticancer activity of the drug was determined in human glioblastoma in several animal experiments. This effect was also found when treating Lewis carcinoma with GcMAF-RF in combination with the Karanahan approach (unpublished data). An analysis of the suppressor activity of tumor lysates obtained after the Karanahan therapy showed that co-administration of Karanahan and GcMAF-RF drugs makes it possible to retain the activated M1 phenotype of murine PMs. Using the established time parameters, the state of the populations of the spleen, tumor immune cells, and blood MNCs was studied starting from the instant of tumor lysis. The results of two independent experiments showed that treating mice engrafted with Lewis carcinoma using the Karanahan approach and GcMAF-RF results in a prominent additive effect, which enhances primary activation of the immune response by the Karanahan therapy (increased number of T helper cells, NKs, CD8 + Perf + cytotoxic T lymphocytes, and regulatory T lymphocytes). GcMAF-RF-activated PMs are also involved in the antitumor immune response.

The results of the MDSC population analysis were inconsistent. The first experiment demonstrated an abrupt increase in the percentage of CD11b + LycC+ and CD11b + LycG + populations of TAS cells in the Karanahan and Karanahan + GcMAF groups. In contrast, a repeated analysis showed a significant decrease in these cell populations in the Karanahan + GcMAF group. It is possible that a large number of leukocytes/neutrophils (purulent discharge) were present in the disintegrating tumor in the first case, which is believed to indicate the migration of peripheral cells to the disintegrating TGS as a result of chemotaxis. This fact was noted as an increase in the corresponding cell populations in the tumor. In the second case, there was no purulent discharge, and the number of CD11b + LycC + and CD11b + LycG + cells was significantly reduced in the Karanahan + GcMAF group. The experiments were carried out in different seasons of the year, and it is possible that there was a shift in the biological clock in Lewis carcinoma cells as shown in Krebs-2 cells [33,56], which changed the active migration of neutrophils. Meanwhile, another effect of GcMAF-RF was clearly established: a significant reduction in the population of myeloid suppressors in the tumor, which can be regarded as a positive prognostic sign. Indicative parameters of the Karanahan approach coincide with the indicators of another novel approach, the low-dose metronomic chemotherapy with CP [57], which allows one to completely cure animals with experimentally transplanted tumors. The studies mentioned above showed that low-dose metronomic chemotherapy with CP eradicates tumor-infiltrating suppressors and activates immune responses in experiments. The results of our experiments support this hypothesis. An abrupt drop in the number of cells carrying markers of myeloid suppressors was noted upon treatment with GcMAF-RF in combination with the Karanahan approach, which led to suppression of immune tolerance caused by the humoral action of the tumor and ensured conditions for migration of peripheral lymphocytes to the TGS and complete lysis of the graft. The findings also suggest that CD11b + LycC + and CD11b + LycG + cells of the TAS that were recruited to the TGS have different biological properties: pro-tumor and tumor-reactive effects, respectively.

Thus, the results of the study suggest that inhibiting the suppressor effect of TAS (MDSCs, regulatory T lymphocytes) is the main condition for the manifestation of the antitumor effect of GcMAF-RF. If the tumor has an active protective humoral background, GcMAF-RF contributes to tumor progression and protection against immune surveillance through its anti-inflammatory effect. If the tumor loses the suppressive humoral background, then pro-inflammatory tumor-reactive effects of GcMAF-RF become prevalent.

The findings obtained in this study indicate that GcMAF-RF appropriately used in combination with the Karanahan approach additively enhances the immune antitumor response induced by the Karanahan therapy.

Further research is required to identify the conditions required to confidently use the drug as an anticancer agent.

## 4. Materials and Methods

### 4.1. Mouse Lines

We used 6–7-week-old SCID mice housed in the SPF Animal Facility (Institute of Cytology and Genetics of the Siberian Branch of the Russian Academy of Science), as well as CBA/Lac and C57BL/6 mice housed under non-SPF conditions (mouse facility at the Institute of Cytology and Genetics of the Siberian Branch of the Russian Academy of Sciences). SCID mice were maintained in OptiMice IVC cages (Animal Care Systems, Centennial, Colorado, USA) in sterile housing rooms ventilated with HEPA14-filtered air under standard 14/10-hr light/dark cycle (lights on at 02:00 a.m.) at a constant room temperature of 22 ± 2 °C and relative humidity of 45 ± 15%. CBA/Lac and C57BL/6 mice were kept in groups of 5–10 animals in plastic cages installed in standard mouse housing rooms with free access to food pellets (PK120-1, Laboratorsnab, Moscow, Russia) and water.

### 4.2. Tumor Models

Human glioblastoma U87 and Lewis carcinoma cell lines were received from the cell collection of the Institute of Cytology and Genetics SB RAS. Human breast adenocarcinoma MCF-7 cells were provided by the Institute of Cell Cultures, State Research Center of Virology and Biotechnology “Vector” (Koltsovo, Novosibirsk oblast, Russia). Human B lymphoma cell line, derived from the aspirate of bone marrow cells of a patient diagnosed with multiple myeloma, was obtained from the Research Institute of Fundamental and Clinical Immunology (Novosibirsk, Russia). A detailed description of the cell line is presented in the study [58]. The cells were cultured in DMEM medium supplemented with 40 μg/mL gentamycin sulfate and 10% fetal bovine serum (FBS) (HyClone, Logan, UT, USA) at 37 °C in a 5% CO_2_ incubator (Memmert, USA LLC, Eagle, WI, USA) to 70–80% confluency.

### 4.3. GcMAF-RF Preparation

DBP was isolated from the serum of healthy donors by affinity chromatography on an actin column [50]. Actin, which is used as an affinity ligand, was obtained from rabbit muscle [59]. A polysaccharide matrix for affinity ligand immobilization was obtained from lobster shells according to [60]. A specific reaction of DBP deglycosylation by stimulated lymphocytes obtained from the same donor and treated with lysophosphatidylcholine was applied for converting DBP to GcMAF-RF [2,61,62].

### 4.4. Analysis of Activation of Phagocytic Activity of Peritoneal Macrophages in Mice

The phagocytic function of macrophages was assessed using the technique presented in [40]. Peritoneal macrophages (PMs) (5 × 10^5^ cells/well) of CBA/Lac mice were cultured in RPMI-1640 medium (BioloT, St. Petersburg, Russia) containing 10% FBS (HyClone) and 40 μg/mL gentamicin in 24-well plates for 2–5 h. Next, the medium was changed to RPMI-1640 supplemented with 10% FBS either in the absence (control) or presence of the following activators: 10 μg/mL *E. coli* 0114:B4 lipopolysaccharide (Sigma, St. Louis, MO, USA) as a positive control and either synthesized DBP or GcMAF-RF at a concentration of 5 μg/mL. The cells were incubated in a CO_2_ atmosphere for 3 h. In an experiment on the effect of conditioned medium of malignant B lymphoma cells, the medium of B lymphoma cells was added to experimental groups for 14 h after the activation procedure described above. Magnetic beads (Dynabeads M-280, Invitrogen, Carlsbad, CA, USA) were then added to each well at a dose of 60 μg/well. After 30-min incubation, macrophages were washed thrice with PBS to remove uninternalized beads. Macrophages were photographed in transmitted light using an Axio Observer Z1 inverted microscope (Zeiss, Jena, Germany), and the number of internalized beads (IBN) was counted. The phagocytic activity of macrophages was assessed using the formula: IBN = number of internalized beads/number of macrophages. The data of four independent experiments were used for statistical analysis of IBN; a total of 300–500 cells were assessed in each experiment. Cells were counted in several fields located in different parts of the plate well.

### 4.5. Assessment of NO Secretion by PMs in Mice

The NO production rate was determined in seven mice in five replicates by evaluating its accumulation after 3 h of incubation with activators in culture supernatants of PMs using the colorimetric method with the Griess reagent [63]. For this, 100 μL of each supernatant was transferred to a 96-well plate, mixed with an equal volume of the Griess reagent, and incubated at room temperature for 15 min. Optical density at 540 nm was estimated using a multichannel spectrophotometer. The results were compared with a standard calibration curve obtained from serial dilutions of 3 mM sodium nitrite solution.

### 4.6. Activation of Functional Properties of Dendritic Cells by GcMAF-RF

DCs were generated from monocytes (3–5 × 10^6^/mL) of the adherent fraction of peripheral blood MNCs from healthy donors. All the volunteers provided written informed consent for having their blood sampled and used for research purposes. The cells were grown in 6-well plates (TPP, Trasadingen, Switzerland) in RPMI-1640 medium supplemented with 0.3 mg/mL L-glutamine, 5 mM HEPES-buffer, 100 μg/mL gentamicin, and 2.5% fetal calf serum (FCS, BioloT, St. Petersburg, Russia) in the presence of GM-CSF (40 ng/mL, Sigma) and interferon-α (1000 U/mL, Roferon-A, Roche, Basel, Switzerland) in a CO_2_ incubator (Memmert USA LLC, Eagle, WI, USA) at 37 °C. To induce the final maturation of dendritic cells, either 10 μg/mL LPS or GcMAF-RF at a dose of 50, 100, 250, and 500 ng/mL was added on day 4, and the cells were incubated for 24 h.

### 4.7. Macrophage Isolation to Analyze M0 to M1 Polarization of Macrophages by GcMAF-RF

Macrophages were generated from monocytes (3–5 × 10^6^/mL) of the adherent fraction of peripheral blood MNCs from healthy donors in RPMI-1640 medium supplemented with 0.05 mM ß-mercaptoethanol, 2 mM sodium pyruvate, 0.3 mg/mL L-glutamine, 1% solution of essential amino acids (all reagents from Sigma-Aldrich), 100 mg/mL gentamicin in the presence of granulocyte-macrophage colony-stimulating factor (rhGM-CSF, 50 ng/mL, Sigma-Aldrich (Burlington, MA, USA)) with 10% fetal calf serum (FCS, BioloT) in 6-well plates (TPP) at 37 °C and 5% CO_2_ for 7 days. Either 10 μg/mL LPS or GcMAF-RF at a dose of either 50 or 250 ng/mL, which was added 48 h before the end of incubation, was used as M1 polarizing signals. GM-CSF-differentiated M0 and polarized M1 macrophages were dissociated from the plastic substratum using 0.25% trypsin-versene solution (BioloT), washed, and then cytosis and viability were assessed.

### 4.8. Assessment of the Allostimulatory Activity of Macrophages and DCs

The stimulatory activity of macrophages and DCs was assessed in a mixed allogeneic culture of leukocytes using donor’s allogeneic MNCs (10^5^/well) as responding cells, which were cultured in RPMI-1640 medium in the presence of inactivated 10% FBS (HyClone) in 96-well round-bottom plates in a CO_2_ incubator (Memmert USA LLC) at 37 °C. Either macrophages or DCs (10^4^ cells) were used as stimulants. The proliferative response was assessed on day 5 radiometrically by incorporation of 3H-thymidine (1 μCi/well), which was introduced 18 h before the end of incubation. The impact index (II) of macrophages and DCs in a mixed allogeneic culture of leukocytes was calculated as the ratio between the proliferative response of MNCs in the presence of macrophages and the level of spontaneous proliferation of MNCs.

### 4.9. Cytokine Production by Whole Blood Cells

To determine the level of cytokine production by human whole blood cells ex vivo, peripheral blood samples from three donors and two separate isolates of GcMAF-RF preparations (i.e., a total of six variants) were used. A 2-mL blood sample was added to a vial containing 8 mL of sterile maintenance medium (DMEM), heparin (2.5 U/mL), gentamicin (100 μg/mL), and L-glutamine (0.6 mg/mL) under sterile conditions. The resulting diluted blood was used to assess the spontaneous production of cytokines and evaluate the effect of GcMAF-RF at final concentrations of 0.05, 0.5, and 5 μg/mL on cytokine production.

Blood samples containing activators were incubated at 37 °C for 24 h. After the end of incubation, blood cells were precipitated by centrifugation in a microcentrifuge at 10,000× *g* for 3 min. The collected supernatant was aliquoted, frozen, and stored at −40 °C for further analysis. The concentrations of IL-2, IL-4, IL-6, IL-8, IL-10, IL-18, IL-1β, TNF-α, IFN-γ, VEGF, and MCP-1 in the supernatant were determined by ELISA using JSC “Vector-Best” reagent kits (Novosibirsk, Russia); the concentrations of G-CSF, GM-CSF, IL-17, and TGF-β were determined using R&D Systems reagents (Abingdon, UK).

The II was used to quantitatively characterize the effect of activators on cytokine production. II = (cytokine production pg/mL in a vial containing diluted blood + drug)/constitutive production of this cytokine (in a vial containing diluted blood without drug). II characterizes the effect of the drug on inactivated blood cells, which mimics GcMAF-RF administration to the patient.

### 4.10. MTT Assay

Direct cytotoxicity of GcMAF-RF was assessed by treating human breast adenocarcinoma MCF-7 and human glioblastoma U87 cells (4 × 10^4^/well) with a GcMAF-RF preparation at a concentration of the target protein of 25 μg/mL. In the case of macrophage-mediated cytotoxicity of GcMAF-RF, murine PMs (4 × 10^4^/well) were preliminarily exposed to 25 μg/mL GcMAF-RF for 3 h, and then tumor cells (4 × 10^4^/well) were added. The survival rate of control cells (without activators) was considered 100%.

In the experiments on immune response assessment, samples of mouse immune cells, namely blood MNCs, splenocytes, and PMs (0.5 × 10^6^ cells), were incubated with Lewis carcinoma cells (10^6^). The survival rate of Lewis carcinoma cells cultured separately in the medium was considered 100%.

The cells were co-incubated in a CO_2_ atmosphere for 24 h. A freshly prepared MTT solution was then added to the cells until the final concentration of 0.5 mg/mL, and the cells were incubated in a CO_2_ incubator (Memmert USA LLC) for 2–3 h. After incubation, the plate was centrifuged at 400× *g* for 5 min, the supernatant was removed, and the crystals were dissolved in 500 μL of dimethyl sulfoxide (VWR Life Science AMRESCO, Solon, OH, USA). Aliquots of 100 μL were taken into a 96-well plate (four replicates for each sample), and the optical density was measured at 550 nm using a Victor X4 plate analyzer (PerkinElmer, Boston, MA, USA) by subtracting the background optical density at 616 nm.

### 4.11. Subcutaneous Grafting of U87 Cells and Assessment of GcMAF-RF Antitumor Effect

SCID mice subcutaneously grafted with U87 glioblastoma cells were used in the experiment. A total of 1 × 10^6^ U87 cells diluted in 100 μL of DMEM were inoculated subcutaneously in the right scapular area of mice. When tumors reached the volume of 0.02 cm^3^, GcMAF-RF injections started. A total of 19 injections of the drug were made once a day. Intraperitoneal and intratumoral injections of the drug were alternated. The drug dose was 2 μg/mouse during the first 14 days and 0.5 μg/mouse during days 15 to 19 (Figure S2, Supplementary Data S2). The volume of intraperitoneal injections was 100 μL; the drug was mixed with DMEM at a ratio of 1:1. The tumor was injected with a medium-free drug at a volume of 50 μL/injection. Mice were examined 2–3 times per week; tumor volume was calculated using the following formula: length × width^2^ × 0.5 [64].

### 4.12. Exposure of Mice to GcMAF-RF Using the Karanahan Approach

A total of 2 × 10^6^ Lewis carcinoma cells were inoculated intramuscularly into the right paw of C57BL/6 mice. Three groups of mice were formed: Control Group; Karanahan Group (animals that received CP and DNAmix injections); and Karanahan + GcMAF-RF Group (animals that received Karanahan therapy with additional administration of GcMAF-RF).

When the tumor reached a volume of 64–340 mm^3^, mice were injected with the drugs. The Karanahan Group was injected with 100 mg/kg of CP intraperitoneally and 0.5 mg/mouse of DNAmix in 200 μL of saline intratumorally according to the therapeutic regimen (Figure 6A) [45]. The production of DNAmix is described in detail in [32]. The Karanahan + GcMAF-RF Group received similar injections with intratumoral administration of 2 μg/injection of GcMAF-RF three hours before each CP treatment (Figure 6B). The Control Group received similar injections of saline. After the therapy initiation, the tumors were measured using a caliper, and the tumor volume was calculated as the product of three measurements (h × l × w).

### 4.13. Effects of Intact Lewis Carcinoma Lysates and Tumor Lysates Obtained after Karanahan Therapy on Murine PMs Activated by GcMAF-RF

Tumor lysates were obtained by homogenizing tumor tissue in lysis buffer containing protease inhibitors. Lysis buffer composed of 50 mM Tris–HCl (pH = 8), 1 mM ETDA, and 100 mM NaCl was added at a ratio of 1 mL of buffer per 0.5 g of tumor. Protease inhibitors were added to the lysis buffer at a ratio of 1/100 V: 0.1 M PMSF, 75 mM TPCK, and 100 mM N-ethylmaleimide. After homogenization, the tumor tissue was sonicated. The resulting lysate was centrifuged at 4000× *g* and 4 °C for 15 min. The supernatant was collected and stored at −70 °C.

To assess the interaction between cancer lysates and GcMAF-RF-activated PMs, the latter (5 × 10^5^ cells/well) were placed in a 24-well plate and then exposed to 5 μg/mL GcMAF-RF 12 h later for 5 h. The cells were then washed and placed in 450 μL of RPMI-1640 medium supplemented with 10% FBS and 40 μg/mL gentamicin; untreated Lewis carcinoma lysates (50 μL/well) and Lewis carcinoma lysates isolated on day 7 after the initiation of Karanahan therapy were added for 16 h. In the regimens including pretreatment of PMs with tumor lysates, the latter (50 μL/well) were added to PMs, which were seeded 12 h before, for 5 h. Macrophages were then washed with PBS thrice by plate centrifugation, and 5 μg/mL GcMAF-RF in RPMI-1640 medium supplemented with 10% FBS and 40 μg/mL gentamicin was added for 18 h (Figure S3, Supplementary Data S2).

To enhance the ability of PMs to resist the devastating effect of the treated tumor lysates, a regimen was developed, which included different variants of preliminary long-term activation of PMs by GcMAF-RF and treatment of activated PMs with the Lewis carcinoma lysate, obtained 7 days after the initiation of the Karanahan therapy. PMs were treated with 5 μg/mL GcMAF-RF either three, five, or six times (Figure 5D). PMs were treated with GcMAF-RF for three days with daily replacement of the drug, washed with PBS, and incubated in RPMI-1640 medium containing 10% FBS for 24 h. Then, activated PMs were co-incubated with 50 μL of tumor lysate in RPMI-1640 medium supplemented with 10% FBS and 40 μg/mL gentamicin for 10 h. GcMAF-RF was added together with the tumor lysates in the case of six-time treatment with the drug. Next, PMs were washed with PBS and incubated in RPMI-1640 medium containing 10% FBS and 40 μg/mL gentamicin for 4 h. PMs were either treated again with GcMAF-RF (in the case of five- and six-time treatment) for 2 days or incubated in RPMI-1640 medium supplemented with 10% FBS and 40 μg/mL gentamicin (in the case of three-time treatment).

The phagocytic activity of macrophages was assessed.

### 4.14. Analysis of Changes in the Number of Immune Cell Populations in the Tumor, Spleen, and among Blood MNCs and PMs

An important note. In this part of the study, we compare the synergistic additive effect of Karanahan therapy in combination with GcMAF-RF to the results of activating the immune response in the group consisting of tumor-bearing mice and the group of mice treated using the Karanahan approach, which were analyzed independently in a separate study. The data are presented in this way because all these three groups (tumor-bearing mice, tumor-bearing mice treated using the novel approach, and tumor-bearing mice treated using the novel approach in combination with GcMAF-RF) simultaneously participated in a series of homotypic experiments. The results of the experiments describing the effect of Karanahan monotherapy and Karanahan therapy in combination with GcMA-RF were reported in two separate studies without any doctrinal interconnection. The first study characterized the efficacy of Karanahan therapy using the Lewis carcinoma model in four replicates. The seasonal and yearly differences in the efficacy of the therapeutic approach were demonstrated. This study also characterized the effect of immune response induction in mice treated using the novel approach (the first two groups were analyzed) [33]. The present study places a key emphasis on characterizing properties of GcMAF-RF: it is shown to exhibit an additive antitumor effect when used in combination with the Karanahan approach and one of its characteristics is the potentiation of the antitumor immune response induced by the novel Karanahan therapy.

To assess the immune response after the Karanahan therapy used in combination with GcMAF-RF, the tumor, spleen, blood MNCs, and PMs were isolated on days 15, 17, and 22 after treatment initiation. TAS cells, populations of spleen immune cells, blood MNCs, and PMs were analyzed using sets of specific antibodies. We compared: (1) the cell populations between the groups (treated and untreated mice, mice treated using the Karanahan approach, and mice receiving Karanahan therapy in combination with GcMAF) at different time points (changes in the activity of the studied cell population in the time interval); (2) the same population of cells in different analyzed anatomical structures (tumor vs. spleen; tumor vs. PMs) in the same group and between the groups; and (3) the state of cell populations depending on the presence or absence of residual tumor tissue was analyzed. Cytolytic antitumor activity of PMs against Lewis carcinoma cells was assessed in separate experiments.

To analyze the populations, the cells were fixed in an equal volume of 4% paraformaldehyde and permeabilized with 0.1% Triton X-100 (VWR Life Science AMRESCO). To block nonspecific binding, PBS containing 10% fetal bovine serum was added to all cells and incubated for 10 min at room temperature. The studied cells (2 × 10^5^) were incubated with 0.25 μg of antibodies and isotype controls at room temperature in the dark for 30–60 min. The following antibodies were used (BioLegend, San Diego, CA, USA, cat. No. is presented in parentheses): suppressor cells of myeloid origin—APC anti-mouse/human CD11b Antibody (101212), PE anti-mouse Ly-6G Antibody (127607), and FITC anti-mouse Ly-6C Antibody (128005); regulatory T lymphocytes—APC anti-mouse CD4 Antibody (100412), FITC anti-mouse CD25 Antibody (101907), and PE anti-mouse FOXP3 Antibody (126403); CD8 + Perf+ cytotoxic T lymphocytes—FITC anti-mouse CD3 Antibody (100204), PE anti-mouse CD8b Antibody (126607), and APC anti-mouse Perforin Antibody (154303); T helper cells—FITC anti-mouse CD3 Antibody (100204) and APC anti-mouse CD4 Antibody (100412); natural killer cells—PE anti-mouse CD335 (NKp46) Antibody (137603), FITC anti-mouse CD3 Antibody (100204), and APC anti-mouse/human CD11b Antibody (101212); dendritic cells—FITC anti-mouse CD80 Antibody (104705) and APC anti-mouse CD83 Antibody (121509). The following isotype controls were used (BioLegend, cat. No is presented in parentheses): APC Rat IgG2b, κ Isotype Ctrl (400612), PE Rat IgG2a, κ Isotype Ctrl (400508), FITC Rat IgG2c, κ Isotype Ctrl (400705), FITC Rat IgG2b, κ Isotype Ctrl (400606), PE Rat IgG2b, κ Isotype Ctrl (400608), APC Rat IgG2a, κ Isotype Ctrl (400511), FITC Armenian Hamster IgG Isotype Ctrl Antibody (400905), APC Rat IgG1, and κ Isotype Ctrl Antibody (400411).

FACS analysis was carried out on a BD FACSAria III cell sorter at the Center for Collective Use of Flow Cytofluorometry of the Institute of Cytology and Genetics of the Siberian Branch of the Russian Academy of Sciences.

### 4.15. Statistical Analysis

Statistical analysis was performed using the Statistica 10 software (StatSoft, Tulsa, OK, USA). The graphs were designed either using Microsoft Excel 2013 (Microsoft, Redmond, Washington, USA) or Statistica 10 (StatSoft, Tulsa, OK, USA), or GraphPad Prism 9.3.1 (GraphPad Software, San Diego, CA, USA) software. The graphs show Mean ± Standard Deviation (SD) or Median with interquartile range values (indicated in the figure legends). The validity of differences was evaluated using either the Mann–Whitney *U* test or an analysis of four-field contingency tables. The revealed differences were considered statistically significant at either *p* < 0.05 (Mann–Whitney *U* test) or χ^2^ *Pv* < 0.01 (analysis of four-field contingency tables). 

## Figures and Tables

**Table 1:** Analysis of secreted cytokines contained in the conditioned medium of B lymphoma cells.

Cytokine (pg/mL)		Conditioned Medium of B Lymphoma Cells		
		Day 3	Day 14	Day 17
Pro-inflammatory	IL-1β	0.9	2.2	0.0
	INF-α	38	40	20
	IL-17	1	1	2
	IL-18	2	2	1
	IL-12	6	6	5
	sTNF	236	247	298
	IFN-γ	1	1	1
	IL-6	30	38	31
	TNF-α	152	110	56
Anti-inflammatory	IL-4	1.4	1.5	1.3
	IL-10	27	32	62
Growth factors	GM-CSF	138	126	86
	G-CSF	31	24	9
	VEGF	1.436	1.353	460
	IL-2	5	5	5
Chemokines	IL-8	1.199	1.241	1.560
	MCP	3.078	3.149	2.567

## Data Availability

The data supporting the findings of this study are available from the corresponding author upon reasonable request.

## Supplementary Materials

The following supporting information can be downloaded at: https://www.mdpi.com/article/10.3390/ijms23158075/s1, References [55,65,66,67,68,69,70,71,72,73] are cited in the supplementary materials.
