# Supplementary material for: Analysis of the Biological Properties of Blood Plasma Protein with GcMAF Functional Activity
Source: Int J Mol Sci. 2022 Jul 22;23(15):8075. doi: 10.3390/ijms23158075 (PMC9330714; doi:10.3390/ijms23158075)
Supplement: Supplementary file 1 [file ijms-23-08075-s001.zip › ijms-1765786-supplementary.pdf]

Supplementary Data S1

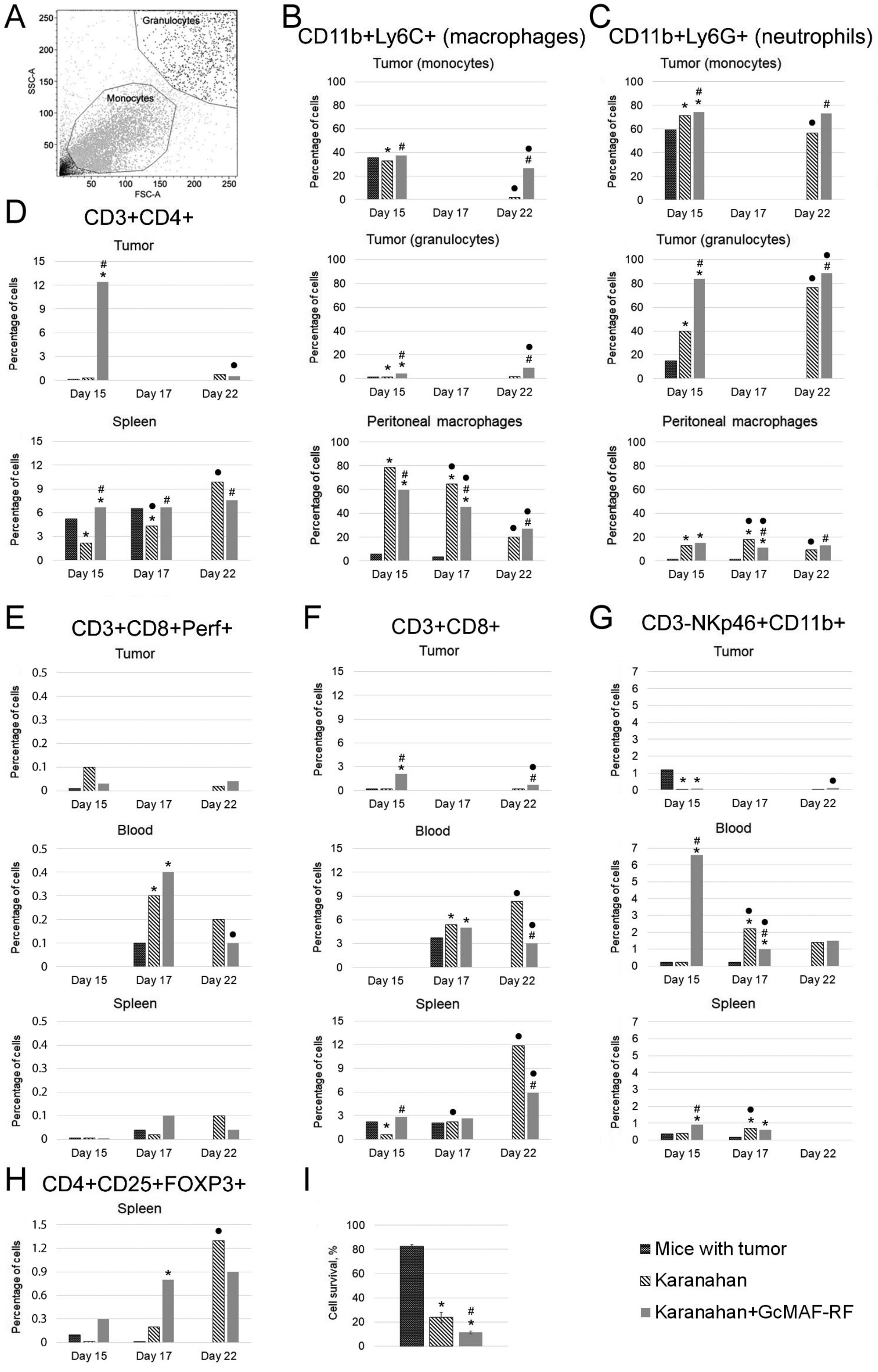

Figure S1. Evaluation of the immune response in mice after the Karanahan and Karanahan+GcMAF-RF therapies. **The primary immunogram**. (**A**) FACS plot. (**B**) CD11b+LyC+ cells. **C**) CD11b+LyG+ cells. (**D**) CD3+CD4+ cells. **E**) CD3+CD8+Perf+ killer T cells. (**F**) CD3+CD8+ cells. (**G**) CD3-NKp46+CD11b+ cells. (**H**) CD4+CD25+FOXP3+ cells. (**I**) MTT assay evaluating the survival of Lewis

carcinoma cells co-cultured with PMs isolated from control animals bearing a tumor and mice after the Karanahan monotherapy and the Karanahan therapy in combination with GcMAF-RF on day 17 after therapy initiation. The survival rate of Lewis carcinoma cells (in the absence of treatments) is considered 100%. Means ± SD values are presented. An asterisk shows a significant difference between the experimental group and mice with tumor, a hash shows a significant difference between two experimental groups, a black circle indicates a significant difference between the results obtained on a certain day and the result of the previous day of sample collection. The confidence level is χ^2 Pv < 0.01 for all comparisons. The results of analyzing the control group of tumor-bearing mice on day 22 are not reported, since all mice either had died because of the tumor by that time or had been withdrawn from the experiment for ethical considerations.

Myeloid-derived-suppressor cells (MDSCs)

The population of suppressor TAS cells includes myeloid progenitors that are believed to determine the pro-tumor status of immune cells infiltrating the tumor site [55,65]. This cell population inhibits maturation of DCs, suppresses the activity of natural killer (NK) cells, and activates the pro-tumor M2/H2 phenotype of tumor-associated macrophages (CD11b+LycC+) and tumor-associated neutrophils (CD11b+LycG+). In Lewis carcinoma, this population is represented by monocytic and granulocytic cells carrying the specific CD11b marker [66].

We compared subpopulations comprising monocyte and granulocyte fractions and determined the number and ratio of CD11b+LycC+ macrophages and CD11b+LycG+ granulocytic neutrophils in the tumor in control and experimental animals. We also evaluated these markers in the general PM population. The following results were obtained.

Distinct monocyte and granulocyte cell populations were clearly present in the tumor (Figure S1A).

Tumor, monocyte fraction

On day 15 of the experiment, the number of CD11b+LycC+ macrophages was approximately 35% in both groups. The number of cells having this phenotype dropped abruptly to its minimal percentage (1%) in the Karanahan Group on day 22 (Figure S1B). The number of CD11b+LycG+ cells also decreased, although less significantly, from 72 to 58% (Figure S1C). The number of CD11b+LycG+ cells in the Karanahan + GcMAF-RF Group decreased insignificantly to ~28% on day 22. The number of CD11b+LycG+ cells in the analyzed subpopulation virtually did not change and remained ~75%.

Tumor, granulocyte fraction

The number of both populations was higher than that in the Control Group. The number of CD11b+LycC+ cells in the Karanahan Group increased from 0.5% to 1.75%, while the number of CD11b+LycG+ cells increased from 40% to almost 80% during the study period. An abrupt increase from 4.2% to 9% in CD11b+LycC+ macrophages was observed in the Karanahan + GcMAF-RF Group. The population of CD11b+LycG+ neutrophils reached 85% on day 1 of evaluation and was further increased to 90% on day 22. An abrupt increase in the number of neutrophils is believed to indicate a pronounced chemotaxis of these cells to the TGS. The results of this experiment indicate that GcMAF-RF sequentially increases the number of granulocytes in the population of CD11b+LycC+ cells at two time points. The subpopulation of granulocytes in the population of CD11b+LycG+ neutrophils reached ~ 40% in the Karanahan Group and 85% in the Karanahan + GcMAF-RF Group *vs.* 10% in the Control Group by day 15. The granulocytic subpopulation further increased to 78% and 90% in the Karanahan and Karanahan + GcMAF-RF Groups, respectively, by day 22. This fact and data on the nature of tumor lysis suggest that GcMAF-RF can induce the large-scale emergence of tumor-reactive neutrophils in the tumor. It is noteworthy that tumor-associated macrophages and tumor-associated neutrophils with CD11b+LycC+ and CD11b+LycG+ phenotypes, respectively, are not always pro-tumor. Arginase activity of these cells, in addition to specific surface markers, is of paramount importance for identifying M1/M2 and H1/H2 phenotypes [67,68].

T helper cells

Mature CD3+CD4+ T helper cells are cells that stimulate and enhance initial immune responses [69,70]. This population of cells has the following features. No significant changes in the cell number were observed in the Karanahan group on days 15 and 22: the cells constituted 0.9% and 2.7% of the population on days 15 and 22, respectively, *vs.* 0.5% in the control on day 15 (Figure S1D). A gradual increase in the number of CD3+CD4+ cells from ~ 2% on day 15 to ~ 4% on day 17 and up to ~ 11% on day 22 was observed in the spleen. The percentage of this type of cells in the Karanahan + GcMAF-RF Group reached 12% on day 15 and reduced to 1.4% on day 22. No significant changes in this parameter were found in the spleen: cell percentage was between 5 and 7%. This cell type is responsible for secondary activation and maturation of effector cells.

CD8+Perf+ cytotoxic T lymphocytes

This is a population of major antigen-specific cytotoxic immune cells. Injection of a dsDNA into TGS and the corresponding stimulation of antigen-presenting properties of DCs of TAS due to the tumor-destroying effect of CP is believed to result in *in situ* priming of numerous naïve CD8+ lymphocytes [71]. This should trigger a T cell response against the vast majority of tumor antigens. After migration and amplification in the spleen, CD8+Perf+ T cells trigger an adaptive antitumor immunity. We assessed the number of tumor, spleen, and blood MNCs and obtained the following results. The percentage of CD3+CD8+Perf+ cells in the tumor was ~0.1% in the Karanahan Group *vs.* 0.01% in the control group. This cell population was evaluated in MNC population and spleen on day 17 and comprised 0.3% *vs.* 0.1% in the control and 0.02% *vs.* 0.04% in the control, respectively (Figure S1 E). On day 22, the percentage of cytotoxic T lymphocytes in the analyzed group decreased to 0.01%; the percentage of CD3+CD8+Perf+ cells decreased to 0.2% in the MNC fraction and increased to 0.1 % in the spleen. The following values were obtained in the Karanahan + GcMAF-RF Group. The number of CD3+CD8+Perf+ cells in the tumor was ~0.03% *vs.* 0.01% in the control on day 15. This cell population was estimated in the mononuclear fraction on day 17 and comprised 0.4% *vs.* 0.1% in the control. An abrupt increase in the number of these cells up to 0.1% *vs.* 0.04 in the control was noted in the spleen. On day 22, the percentage of cytotoxic T lymphocytes increased to 0.04% in the tumor; the content of CD3+CD8+Perf+ cells decreased to 0.1% and 0.04% in the fraction of MNCs and the spleen, respectively. An analysis performed between the experimental groups indicated the multidirectional nature of changes in the percentage of cytotoxic T lymphocytes in the tumor and spleen and the unidirectional nature of changes in blood MNCs. The number of cytotoxic T lymphocytes on day 15 in the Karanahan Group drops on day 22 in the tumor (Figure S1E). There was an increase in CD3+CD8+Perf+ cells by day 22 in the Karanahan + GcMAF-RF Group. In the spleen, on the contrary, there was a fivefold increase in this parameter in the Karanahan Group from day 17 to day 22. Cell percentage decreased significantly by day 22 in the Karanahan + GcMAF-RF Group. These changes indicate that the rate of killer cell production in the tumor in the Karanahan Group was higher than that in the Karanahan + GcMAF-RF Group. In the spleen, on the contrary, a higher percentage of CD3+CD8+Perf+ cells was observed in the Karanahan + GcMAF-RF Group compared to the Karanahan Group on day 17, which indicates an earlier onset of adaptive immunity. Evaluating the number of CD3+CD8+ lymphocytes is a matter of interest. These are naive cytotoxic T lymphocytes, and their number also characterizes the onset of adaptive immunity. On day 15, the same percentage of immature CD3+CD8+ cells was present in the tumor both in the control and the experimental animals of the Karanahan Group: ~ 0.2% (Figure S1F). The number of cells did not change for 7 days. The percentage of cells carrying these markers in blood was 6% *vs.* 4% in the control at the second time point and increased to 9 % by the third time point. The following pattern of distribution of the cell population was observed in the spleen: the cell number was 0.6% *vs.* 2.2% in the control on day 15; 2.1% *vs.* 2.2% in the control on day 17; and 12% on day 22. In the Karanahan + GcMAF-RF Group, the percentage of CD3+CD8+ lymphocytes in the tumor was 2% on day 15, which is ten times more than that in the control and Karanahan Group. The percentage dropped to 0.7% by day 22. The number of cells with

the same markers in blood was 5% *vs.* ~ 4% in the control on day 17 and decreased to 3% on day 22. The parameter gradually increased in the spleen and reached 6% by day 22.

Natural killer cells

NK cells are the main population of cells that can lyse a tumor without preliminary antigenic recognition [72]. This population of cells is practically absent in the TGS of treated animals, while the percentage of CD3-NKp46+CD11b+ NKs in the control is 1.2% (supposedly anergic cells). The percentage of NK cells in blood MNCs in the Karanahan Group increased from 0.2% on day 15 to 2% on day 17 and slightly decreased to 1.0 % on day 22 (Figure S1G). The number of mature NKs in splenocytes increased from 0.4 to 0.6% on days 15 and 17, respectively. A significant rise in NK percentage in the peripheral blood to 7% in Karanahan + GcMAF-RF Group was observed on day 15, which is followed by a drop to ~1% on days 17 and 22. The number of NK cells in the spleen reached 0.9% on day 15 and decreased to 0.6% on day 17. A large percentage of NK cells in the blood suggests that they have a prominent anti-tumor effect on disseminating tumor cells.

Regulatory T lymphocytes

CD4+CD25+FoxP3+ regulatory T lymphocytes exhibit a pronounced suppressive activity and normally suppress the immune response after complete neutralization of a pathogen. In case of a malignant neoplasm, regulatory T lymphocytes are recruited to the TGS to induce mechanisms through which the developed neoplasm avoids the immune surveillance [73]. The performed analysis indicates the following. No CD4+CD25+FoxP3+ cells were detected in the TGS in the Karanahan Group during the entire study period. This population of cells was first detected (0.01%) on day 17 and reached 1.2% by day 22 in the spleen of the Karanahan Group (Figure S1 H). The number of cells in the control lay within the range of instrumental error. A different pattern was obtained for the Karanahan + GcMAF-RF Group. A small amount (0.06%) of CD4+CD25+FoxP3+ cells was detected in the tumor on day 22. The percentage of these cells in the spleen gradually increased by 0.3%, 0.7%, and 0.9% on days 15, 17, and 22, respectively. The occurrence of regulatory T lymphocytes in the spleen is indicative of activation of feedback control mechanisms suppressing excessive escalation of activated immune responses in both experimental groups.

Evaluation of cytolytic activity of PMs against Lewis carcinoma cells by the MTT assay

The main indicator of induced antitumor immunity in a particular population of immune cells is their direct cytolytic effect on tumor cells. PMs were analyzed separately as a population of cells forming an immune barrier outside the circulatory system. We performed a series of experiments to determine the effectiveness of the cytotoxic effect of PMs on Lewis carcinoma cells. On day 17, the studied populations of cells and target Lewis carcinoma cells were mixed at a 1:2 ratio and incubated for 24 hrs. The results of MTT assay are presented in Figure S1I. PMs had a pronounced antitumor effect in both experimental groups of mice. According to the results of the MTT assay, about 80% of tumor cells were lysed six days after termination of the Karanahan therapy. Phagocytes isolated from the Karanahan + GcMAF-RF Group of mice had the most prominent antitumor effect. The MTT assay showed that 90% of tumor cells were lysed. No antitumor activity of PMs was detected on day 31 after termination of the Karanahan therapy. A partial cytolytic effect against Lewis carcinoma cells was observed in the Karanahan + GcMAF-RF Group.

Supplementary Data S2

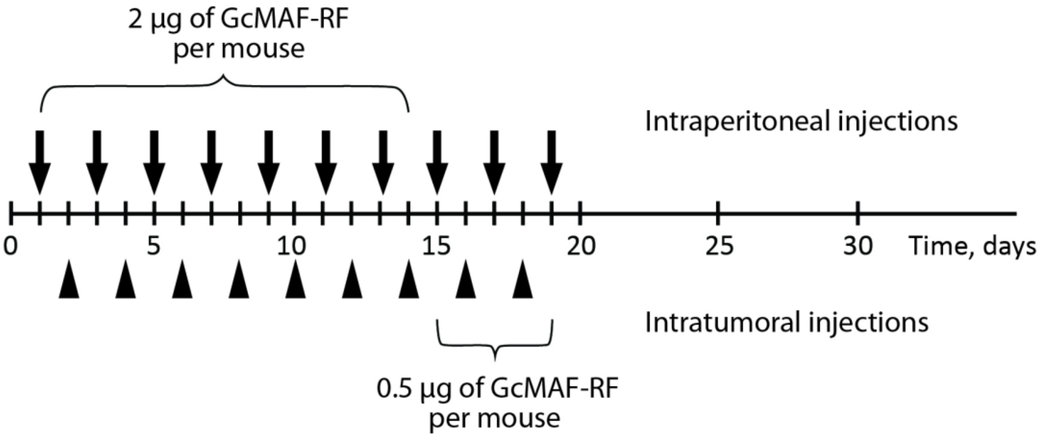

Figure S2. Scheme of GcMAF-RF administering to SCID mice with subcutaneously transplanted U87 glioblastoma tumors. A total of 19 injections of the drug occurred once a day. Intraperitoneal (arrows) and intratumoral (arrowheads) injections of the drug were alternated. The drug dose was 2 µg/mouse during the first 14 days and 0.5 µg/mouse during days 15 to 19.

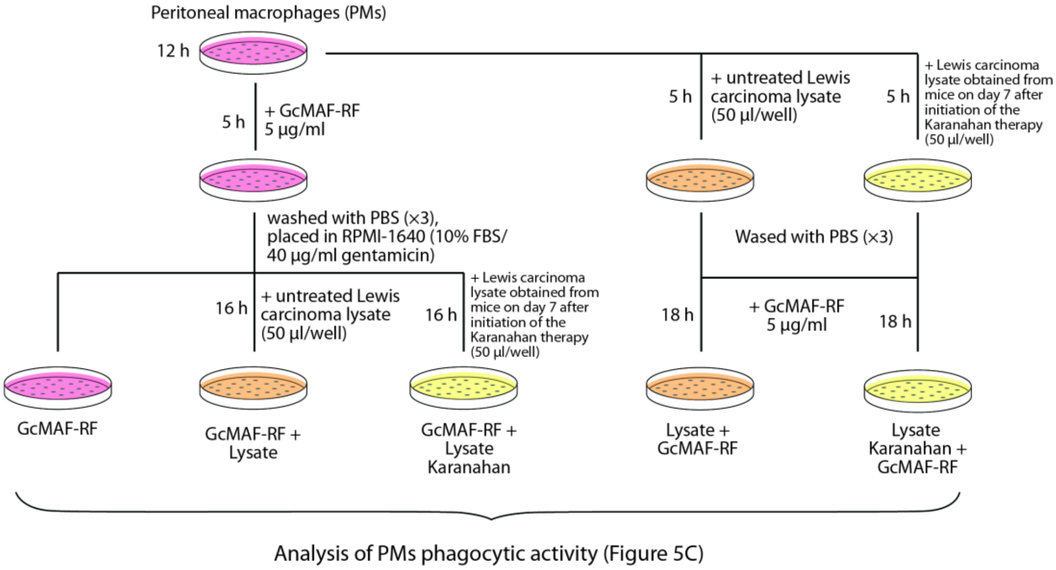

Figure S3. Analysis of the interaction between Lewis carcinoma lysates and GcMAF-RF-activated PMs. Schematic representation of pre-treatments.
